# Acute Aortic Occlusion Mimicking Cauda Equina Syndrome: Complete Neurologic Recovery After Emergent Endovascular Revascularization

**DOI:** 10.7759/cureus.98174

**Published:** 2025-11-30

**Authors:** Nicholas L Todd, Austin Schaff, Stephen Saela, Jacob Roe, Nicole Griffin, Frederick Korpi, Jeffrey Cochran

**Affiliations:** 1 Orthopedic Surgery, Aultman Hospital, Canton, USA; 2 Orthopedics, Aultman Hospital, Canton, USA; 3 Orthopedic Trauma, Spectrum Orthopedics, Canton, USA

**Keywords:** aortic aneurysm, cauda equina, paralysis, spine surgery, vascular surgery

## Abstract

A 67-year-old man with a history of bilateral iliac stenting presented for acute right lower extremity weakness, numbness, and severe pain after exertion. Femoral and distal pulses were absent bilaterally. Initial suspicion during evaluation was for Cauda Equina Syndrome. However, computed tomography angiography of the abdomen and pelvis revealed an acute thrombotic occlusion of the distal abdominal aorta extending into both common iliac arteries with a 3.1-cm aneurysm. Emergency vascular intervention was performed, consisting of over-the-wire thrombectomy and endovascular stenting with adjunctive balloon angioplasty. Blood flow to both lower extremities was restored. On postoperative day one, strength and sensation normalized with palpable distal pulses. The patient experienced complete resolution of lower extremity symptoms and neurologic deficits. This case emphasizes the importance of clinical awareness, rapid diagnosis, and urgent treatment of an acute vascular condition that can mimic cauda equina syndrome.

## Introduction

Acute aortic occlusion (AAO) is a rare but devastating vascular emergency that can masquerade as cauda equina syndrome because its abrupt loss of distal perfusion triggers sudden leg weakness, numbness, and bladder or bowel dysfunction. Delays in recognition can be catastrophic for both life and limb. Although AAO is most commonly associated with thromboembolic events, it may also occur in the setting of chronic atherosclerotic disease or following prior vascular interventions, as seen in patients with pre-existing stents or aneurysms [1,2]. The clinical presentation of AAO can be variable; in some cases, patients present with acute bilateral lower extremity ischemia and neurologic deficits that mimic primary spinal pathologies such as cauda equina syndrome (CES), potentially leading to diagnostic and therapeutic delays [3].

In recent years, literature has emphasized the importance of early recognition and rapid revascularization to prevent irreversible neurologic damage and limb loss [4]. Endovascular therapies have emerged as the primary modality in the management of AAO, offering the benefits of less invasiveness and rapid restoration of perfusion compared to traditional open surgical techniques [5]. This report describes a 67-year-old man with a history of bilateral iliac stenting who presented with a sudden onset of complete right lower extremity paralysis and numbness. Imaging revealed an occlusive process in the distal abdominal aorta extending through the common iliac arteries, compounded by the presence of a small abdominal aortic aneurysm. The patient underwent emergent endovascular intervention, resulting in complete resolution of his neurologic deficits. This case report underscores the necessity of considering vascular causes in the differential diagnosis of acute lower extremity paralysis and highlights the effectiveness of prompt endovascular management.

## Case presentation

A 67‐year‐old man with a history of bilateral iliac stenting (2021) and noncompliance with medical follow-up presented to our facility with an acute onset of right lower extremity weakness and pain, as well as back pain around 9 PM. The patient, who had been performing manual labor (loading wood into a truck and later moving leaves and trees), experienced sudden severe low-back pain radiating down the right leg with associated numbness around 6 PM. He reported he felt a “pop” which incited the symptoms. His friend assisted him into a vehicle following the injury. He then sought medical attention secondary to the significant pain.

The patient reported complete loss of sensation and motor function in his right lower extremity. He noted that the numbness started immediately after the inciting “pop.” His primary complaint in the emergency department was lower back pain. He denied any loss of bowel or bladder function, perianal numbness, or upper extremity symptoms. His left lower extremity demonstrated full motor strength and sensation, although he did report non-dermatomal paresthesias. The patient had no prior history of spine surgery, back pain, or neurologic issues. He denied taking any anticoagulants or antiplatelet medications. He did report a history of tobacco use.

On physical exam, he was alert, oriented, and cooperative. Femoral pulses were non-palpable bilaterally. Doppler signals were absent in both legs at the dorsalis pedis, posterior tibial, popliteal, and femoral arteries. The orthopedic spine team’s evaluation confirmed no upper extremity involvement. Examination of the left lower extremity revealed a full painless range of motion at the hip, knee, and ankle, with normal strength in lumbosacral myotomes. Sensation was intact to light touch over the L3-S1 dermatomes. Deep tendon reflexes at the ankle and knee are absent, and there were zero beats of clonus. Examination of the right lower extremity revealed an absence of sensation in the distal aspect of the leg, with sensation returning proximally at approximately the mid-thigh level. Motor examination revealed no functional movement at the ankle and toes. There was only a minimal "flicker" of quadriceps and hamstring activation. Hip flexion strength was 0/5. As with the left side, deep tendon reflexes at the ankle and knee were absent. No clonus was observed.

**Figure 1:**
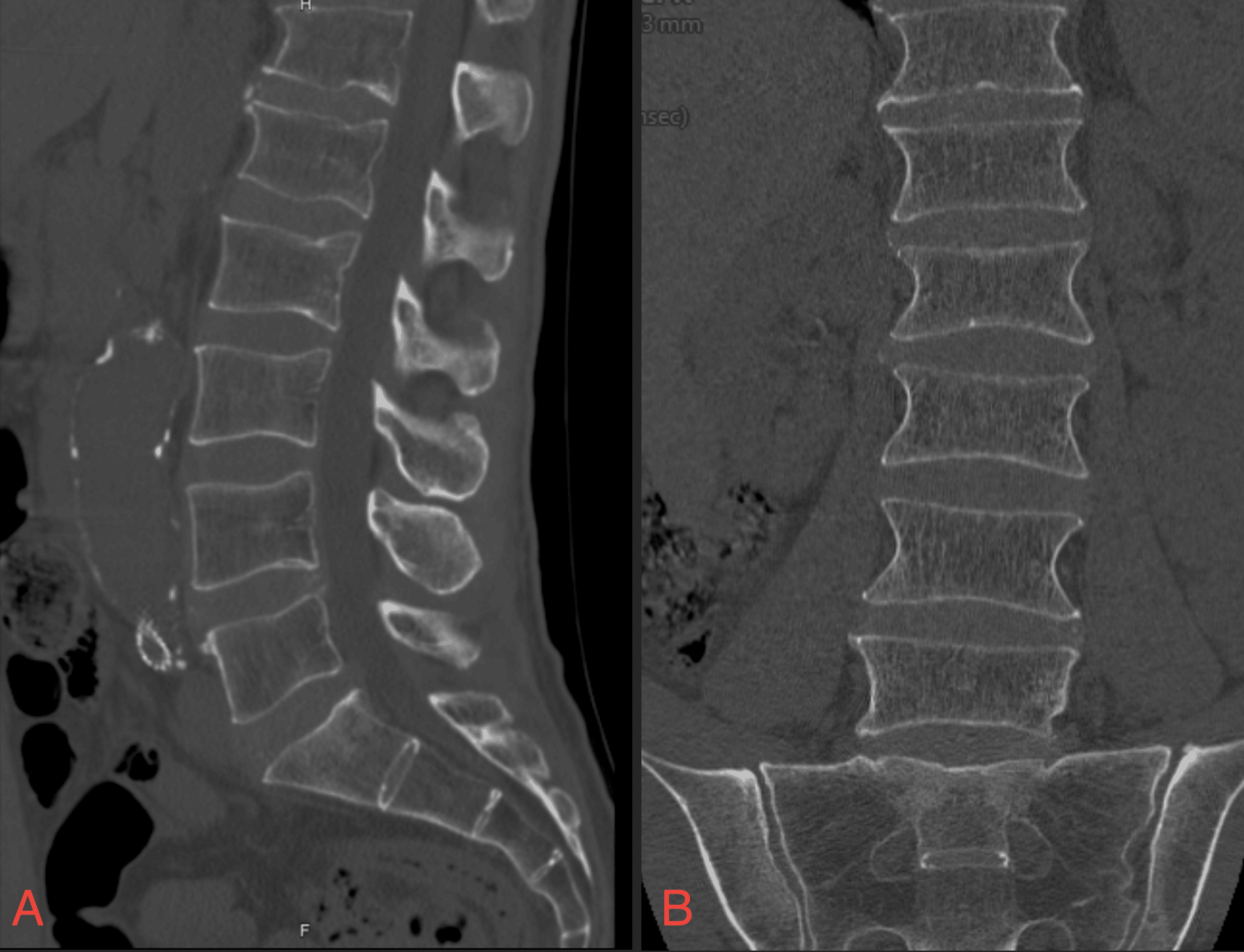
Sagittal (A) and coronal (B) images from CT scan of lumbar spine

A computed tomography (CT) scan of his lumbar spine (Figure 1) demonstrated normal alignment without acute fractures or significant degenerative changes that could account for the neurologic findings, further supporting a vascular etiology.

**Figure 2:**
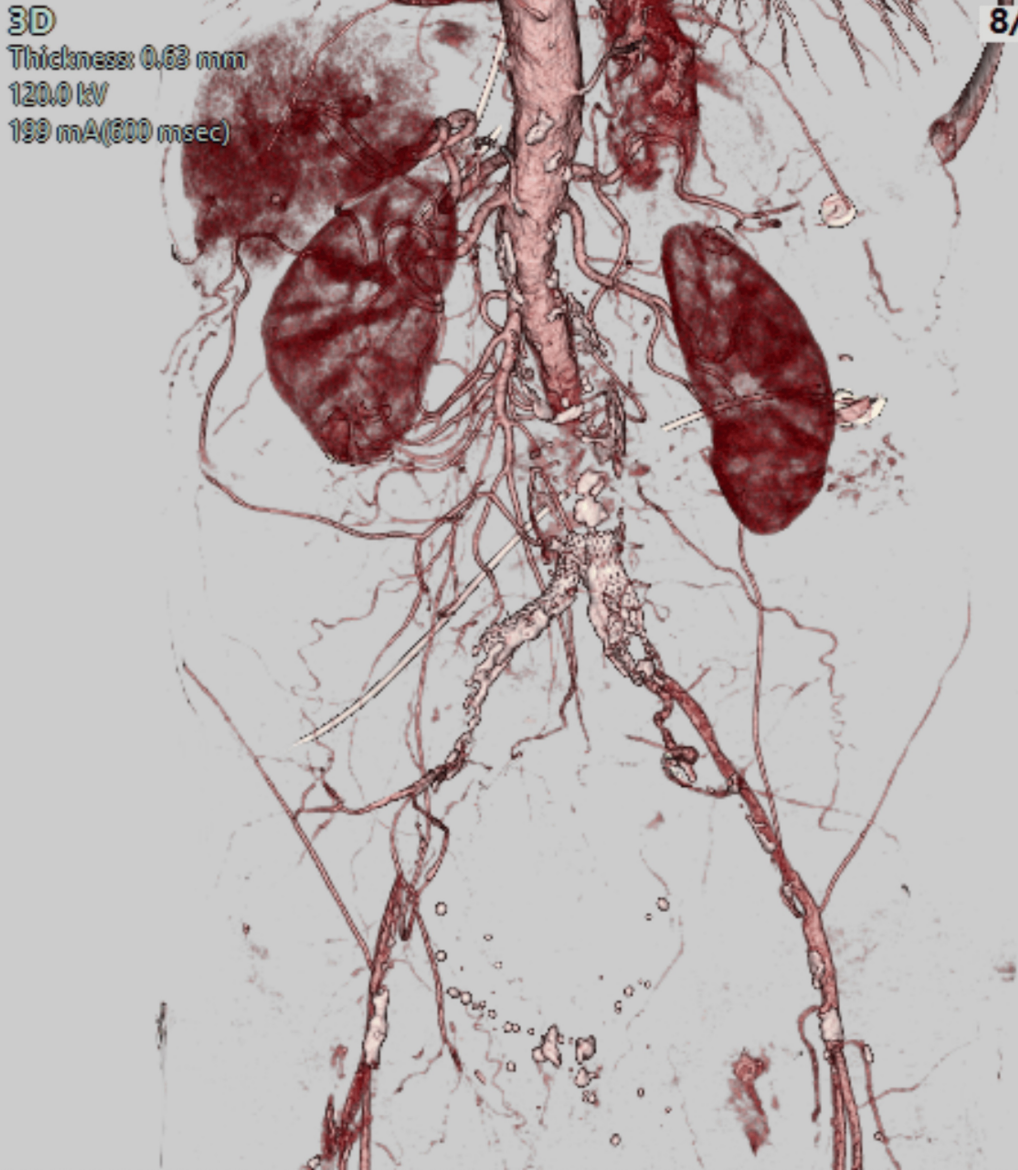
Three-dimensional reconstruction of CTA CTA: computed tomography angiogram

An initial computed tomography angiogram (CTA) of the abdomen and pelvis ordered in the emergency department revealed an occlusive process involving the distal abdominal aorta and bilateral common iliac arteries (Figure 2).

**Figure 3:**
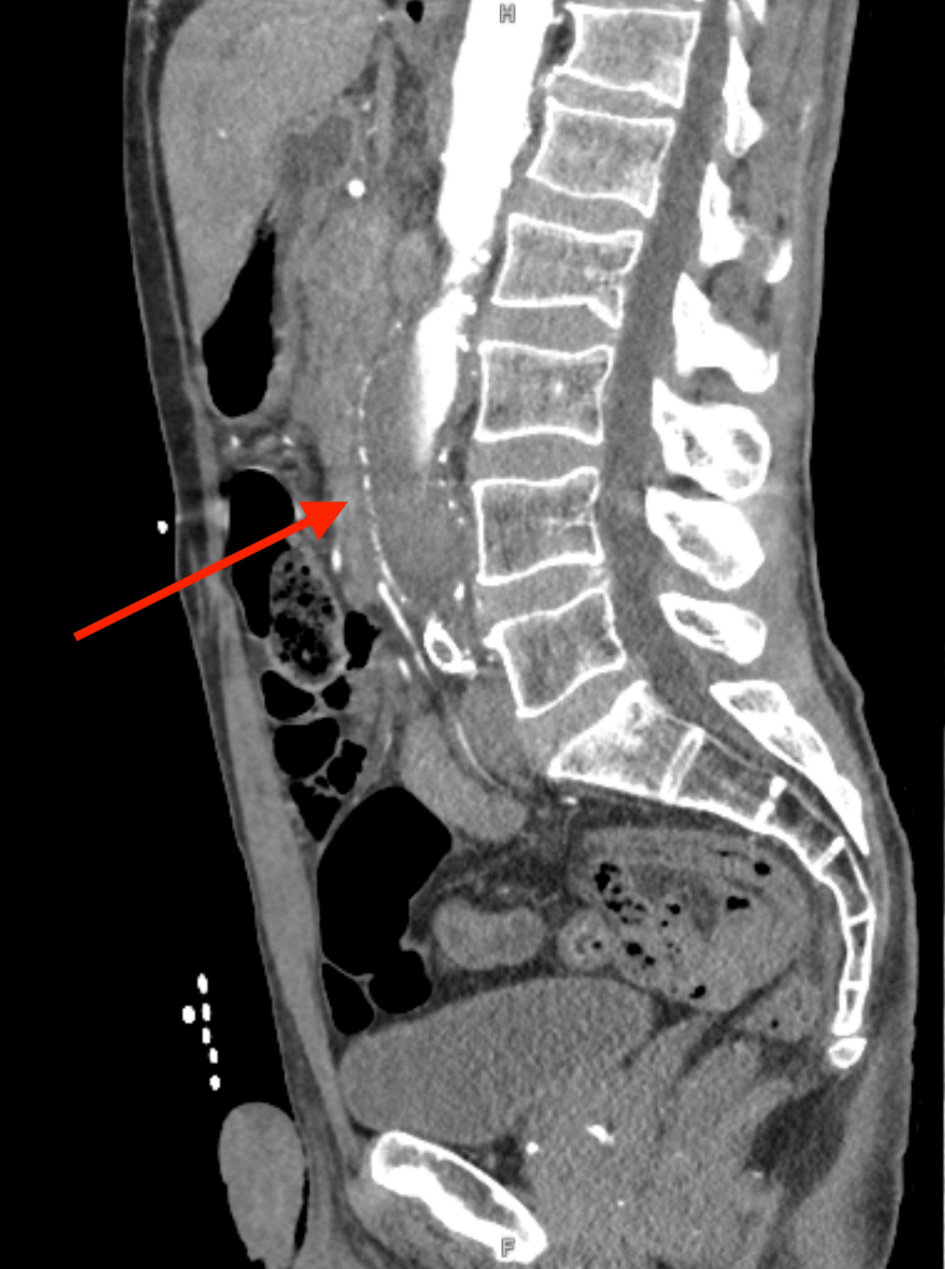
Sagittal image from CTA demonstrating the aneurysm (red arrow) CTA: computed tomography angiogram

The imaging noted a 3.1 cm abdominal aortic aneurysm with delayed opacification of the aneurysm on the arterial phase and reconstitution of flow via collaterals on the portal venous phase in the mid to distal common iliac vessels (Figure 3). The CTA also documented a prior stent graft at the level of the aortic bifurcation extending into the common iliac arteries. MRI was not obtained due to absent femoral/distal pulses and time-critical revascularization needs.

A diagnosis of acute thrombotic occlusion resulting in bilateral lower extremity ischemia, with the right lower extremity manifesting severe neurologic deficits consistent with ischemic injury, was made.

Given the critical findings of vascular insufficiency, the vascular surgery team promptly initiated weight-based heparin therapy and arranged for emergent operative intervention. Under general anesthesia, bilateral common femoral artery access was obtained via open cutdown and Seldinger technique. Systemic heparinization was achieved, and over-the-wire thrombectomy was performed from the iliac limbs into the aorta, removing a substantial amount of thrombus. Intraoperative angiography confirmed the roughened aortic wall and the presence of the aneurysm. Recognizing the need for durable revascularization, an endograft stent was deployed in an infrarenal position with appropriate extension into the iliac systems. Adjunctive balloon angioplasty (including kissing balloon technique at the bifurcation) was performed to optimize luminal diameter and flow. Post-procedure, angiography demonstrated excellent restoration of blood flow through the aorta and into both lower extremities. The femoral arteriotomies were repaired primarily, and the patient was transferred to recovery in stable condition.

On the morning of postoperative day one, the patient exhibited complete resolution of his lower extremity weakness with palpable pulses and pink, well-perfused feet. He did report mild muscle tenderness in the right calf and gluteal region, but the compartments were soft, and there was no pain on passive movement. His overall recovery was uneventful; he tolerated a diet and began ambulating with full resolution of his neurologic deficits. The patient was counseled extensively on the importance of medication compliance, follow-up, and tobacco cessation.

He was discharged home on postoperative day one after meeting all postoperative milestones. He was lost to outpatient follow-up and did not return to the outpatient spine or vascular clinic for re-evaluation.

## Discussion

Acute aortic occlusion (AAO) is a vascular catastrophe that, while uncommon, poses significant morbidity and mortality risks if not rapidly recognized and managed. In this case, the patient presented with neurologic deficits that initially raised concerns for a primary spinal pathology. However, the clinical context, including a history of prior iliac stenting combined with the vascular examination findings of absent femoral pulses, prompted further vascular imaging that revealed occlusion of the distal abdominal aorta extending into the common iliac arteries. It is unclear exactly why our patient thrombosed; possible mechanisms include in-stent thrombosis propagating proximally vs in-situ aortic thrombosis over roughened wall/aneurysmal segment.

The diagnostic challenge in AAO lies in its protean presentation. As noted in the literature, AAO can mimic neurologic emergencies due to the rapid onset of motor and sensory deficits secondary to ischemia rather than nerve compression or injury [1,2]. Misdiagnosis or delays in intervention can result in irreversible ischemic damage, emphasizing the need for a high index of suspicion, especially in patients with known atherosclerotic disease or prior vascular interventions [3]. In our patient, the absence of spinal pathology on CT lumbar spine imaging further supported a vascular etiology. It is paramount to do an adequate pulse exam on patients such as this, including Doppler signals, before imaging of the spine.

Recent advances in endovascular therapy have transformed the management of AAO. Endovascular interventions, such as thrombectomy combined with stent grafting, have demonstrated promising results in the rapid restoration of perfusion while reducing the morbidity associated with open surgical procedures [4,5]. In this case, the use of over-the-wire thrombectomy and subsequent deployment of an endograft resulted in the prompt return of blood flow and complete resolution of the patient's neurologic deficits. This outcome aligns with findings from prior studies, which suggest that early revascularization significantly improves limb salvage and neurologic outcomes [6].

Furthermore, the case underscores the importance of a multidisciplinary approach. Collaboration between vascular surgery and orthopedic spine teams was crucial for comprehensive assessment and timely intervention. Literature supports that such coordinated care in the management of complex vascular emergencies not only expedites diagnosis but also facilitates optimal therapeutic strategies [7].

## Conclusions

This case illustrates the diagnostic challenges associated with acute aortic occlusion mimicking CES. Particularly high index of suspicion must be maintained, especially in patients with prior vascular interventions and noncompliance with medical therapy. A thorough exam of pulses, leading to urgent diagnostic CTA and revascularization in a timely manner, is critical for patient outcomes. The complete neurologic recovery observed following emergent endovascular management highlights the necessity for early vascular imaging and interdisciplinary collaboration. Timely revascularization not only salvaged limb function but also prevented permanent neurologic injury, reinforcing that vascular etiologies must be thoroughly considered in the differential diagnosis of acute lower extremity paralysis.
